# Body Temperature and Activity Rhythms Under Different Photoperiods in High Arctic Svalbard ptarmigan (*Lagopus muta hyperborea*)

**DOI:** 10.3389/fphys.2021.633866

**Published:** 2021-03-08

**Authors:** Daniel Appenroth, Andreas Nord, David G. Hazlerigg, Gabriela C. Wagner

**Affiliations:** ^1^Arctic Chronobiology and Physiology, Arctic and Marine Biology, UiT The Arctic University of Norway, Tromsø, Norway; ^2^Section for Evolutionary Ecology, Department of Biology, Lund University, Lund, Sweden; ^3^Division of Forest and Forest Resources, Norwegian Institute of Bioeconomy Research (NIBIO), Tromsø, Norway

**Keywords:** Arctic, chronobiology, circadian rhythm, heterothermy, photoperiod, thermoregulation, Svalbard ptarmigan

## Abstract

Organisms use circadian rhythms to anticipate and exploit daily environmental oscillations. While circadian rhythms are of clear importance for inhabitants of tropic and temperate latitudes, its role for permanent residents of the polar regions is less well understood. The high Arctic Svalbard ptarmigan shows behavioral rhythmicity in presence of light-dark cycles but is arrhythmic during the polar day and polar night. This has been suggested to be an adaptation to the unique light environment of the Arctic. In this study, we examined regulatory aspects of the circadian control system in the Svalbard ptarmigan by recording core body temperature (*T*_b_) alongside locomotor activity in captive birds under different photoperiods. We show that *T*_b_ and activity are rhythmic with a 24-h period under short (SP; L:D 6:18) and long photoperiod (LP; L:D 16:8). Under constant light and constant darkness, rhythmicity in *T*_b_ attenuates and activity shows signs of ultradian rhythmicity. Birds under SP also showed a rise in *T*_b_ preceding the light-on signal and any rise in activity, which proves that the light-on signal can be anticipated, most likely by a circadian system.

## Introduction

The Earth’s rotation around its own axis causes daily oscillations in environmental factors such as light and ambient temperature. Circadian rhythms have evolved to maintain behavioral, physiological, and metabolic synchrony with these ambient cycles, and to anticipate changing conditions within a day. Disruption of this synchrony can consequently affect fitness and survival (DeCoursey et al., 1997, 2000; DeCoursey and Krulas, 1998; Daan et al., 2011; Spoelstra et al., 2016). Biochemical oscillators, so-called circadian clocks, endogenously produce rhythmicity by transcription-translation-feedback loops, in which clock genes are expressed and subsequently inhibited due to the action of their own translated proteins (Hardin et al., 1990; Darlington et al., 1998). In higher vertebrates, circadian rhythmicity is ultimately produced by a single hypothalamic master clock (the suprachiasmatic nucleus in mammals) or a network of clocks (in the pineal gland, eyes and the hypothalamus of bird and reptiles; Menaker et al., 1997). These master clocks entrain to the environmental cycle primarily through the light-dark signal (Pittendrigh, 1960) and impose rhythmicity onto peripheral tissue, e.g., by circulating hormones such as melatonin produced in the pineal gland (Pevet and Challet, 2011). This ultimately leads to rhythmic physiology and behavior.

**Figure 1:**
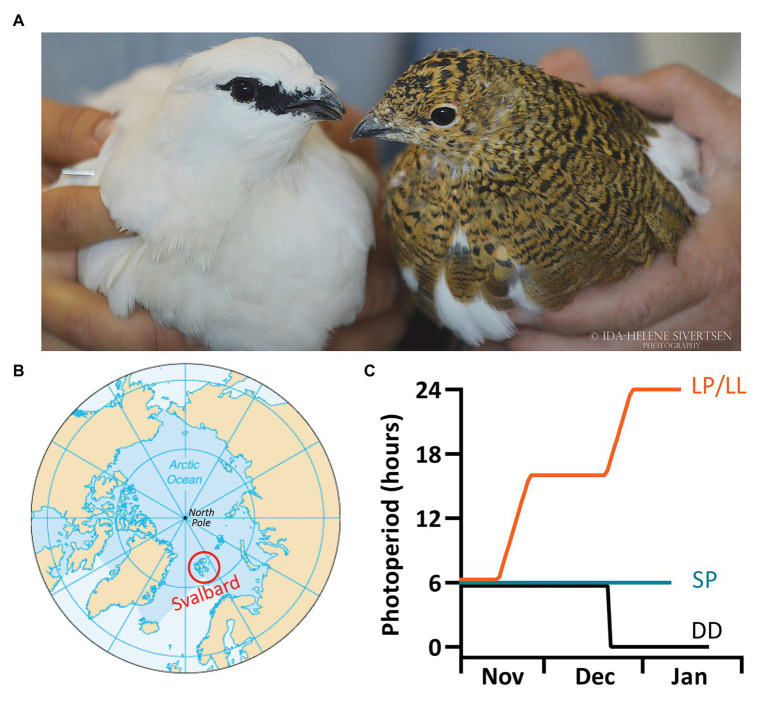
Svalbard ptarmigan (*Lagopus muta hyperborea*) and experimental design. **(A)** The picture shows a Svalbard ptarmigan male in white winter plumage and a female in the cryptic brown summer plumage (© Ida-Helene Sivertsen). **(B)** The Svalbard ptarmigan is a subspecies of the rock ptarmigan (*Lagopus muta*) but is geographically isolated to the high Arctic archipelago of Svalbard and Franz Josef Land. **(C)** The experimental birds were bred at the University of Tromsø and were separated into three groups: the short photoperiod (SP) group remained under L:D 6:18. The LP/LL-group was gradually transferred from L:D 6:18 to L:D 16:8 (LP), and subsequently into constant light (LL). The constant darkness (DD) group was directly transferred from L:D 6:18 into DD.

At tropical and temperate latitudes, the light-dark progression and other environmental factors, such as ambient temperature, cycle on a 24-h period throughout the year. This is not the case at polar latitudes, which are instead characterized by extended periods of constant light (polar day) and constant darkness (polar night) with short periods of rapidly changing photoperiod in-between. During the polar day and polar night, animals inhabiting these latitudes are either free running, arrhythmic, or entrained to non-photic or photic cues other than photoperiod (Swade and Pittendrigh, 1967; van Oort et al., 2007; Stelzer and Chittka, 2010; Williams et al., 2011; Ashley et al., 2012; Steiger et al., 2013; Arnold et al., 2018; Hüppe et al., 2020; van Beest et al., 2020; Ware et al., 2020). On the Svalbard archipelago, which is among the northernmost landmasses in the Arctic (74°–81°N, Figure 1B), the Sun remains ≥6° below the horizon between mid-November and February but is constantly above the horizon between mid-April and mid-September. Despite the high latitude, the climate is relatively mild due to warm ocean currents (average temperature in Longyearbyen in December: −6.0°C; Norwegian-Meterological-Institute, 2020). This is probably why some cold-hardy species can survive there year-round, including the world’s most northerly distributed land bird, the Svalbard ptarmigan (*Lagopus muta hyperborea*; Figure 1A). Svalbard ptarmigan exhibit rhythmic activity during the short periods of light-dark cycles, but become arrhythmic during the polar day and polar night (Stokkan et al., 1986; Reierth and Stokkan, 1998). Similarly, plasma melatonin rhythms attenuate under such conditions (Reierth et al., 1999). This suggests that either the central circadian clock cannot sustain rhythmicity, or that its molecular output is uncoupled from the peripheral tissue responses in constant light and constant darkness (Bloch et al., 2013). The above mentioned studies indicate that adaptation to life in the high Arctic has had profound effects on the circadian system. However, little is known of the physiological parameters that underlie circadian control. For this reason, we explored the implication of Arctic life on the circadian control of core body temperature (*T*_b_).

Most endothermic animals display a daily *T*_b_ rhythm, which is under circadian control and characterized by lower *T*_b_ during the rest phase and higher *T*_b_ during the active phase (Menaker, 1959; Aschoff, 1983; Refinetti and Menaker, 1992). The function of the *T*_b_ rhythm is still disputed. The decrease in *T*_b_ during the rest phase might reduce energy costs by lowering the need for thermogenesis, though in the case of non-torpid and non-hibernating endotherms this reduction in *T*_b_ might be too small to have a significant impact on the energy budget (Menaker, 1959; Refinetti and Menaker, 1992). Temperature changes within the physiological range have also been shown to sustain rhythmicity in mammalian liver and lung cultures (Brown et al., 2002; Buhr et al., 2010) and it has been proposed that *T*_b_ serves the master clock to synchronize peripheral tissue. In a polar animal, *T*_b_ might serve the same purpose, especially since melatonin rhythms are often attenuated under the polar day and polar night (Miché et al., 1991; Reierth et al., 1999; Stokkan et al., 2007). There is evidence of sustained *T*_b_-rhythmicity through the polar day in Arctic ground squirrels (*Urocitellus parryii*; Long et al., 2005; Williams et al., 2011), but we are unaware of similar studies in Arctic birds.

In order to characterize the *T*_b_ rhythm in a truly Arctic bird and explore its possible circadian control, we implanted abdominal temperature loggers into captive Svalbard ptarmigan and recorded *T*_b_ and activity under short photoperiod (SP), long photoperiod (LP), in constant light (LL) and constant darkness (DD; Figure 1C). These photoperiodic treatments were chosen to study expression of the *T*_b_ and activity rhythm under entrained conditions (SP and LP) as well as under conditions without entrainable cues, i.e., in free running conditions (LL and DD). We also studied if there were differences in the timing of the rise in activity and *T*_b_ before the light-on signal when birds were under SP and LP, because this “anticipatory behavior” could indicate the presence of a functional time-keeping system.

## Materials and Methods

### Housing

All animals were kept at the University of Tromsø in accordance with the EU directive 201/63/EU and licenses provided by the Nowegian Food Safety authority (Mattilsynet, permit nos. FOTS 8115 for 2015/2016 and FOTS 7971 for 2017/2018). Chicks were hatched from eggs laid by captive females in 2015 and 2017, and were reared either in outdoor cages under natural Tromsø photoperiod (69° 39′ N, 18° 57′ E), or indoors with a photoperiod corresponding to the natural light cycle in Tromsø. When the chicks had reached a body mass of 500 g or more (usually by the end of September in the year of hatching), they were transferred to indoor cages with *ad libitum* access to food (Norgesfor, ref. no. OK2400 070316) and water. Ambient temperature was kept between 3 and 7°C throughout the experiment, which is within the thermo-neutral zone of physically mature Svalbard ptarmigan (Mortensen and Blix, 1986).

Illumination was provided by fluorescent strip lights (Osram L 58W 830 Lumilux, Osram, Munich, Germany), delivering 1,000 lux at floor level. Under constant darkness, illumination was provided by dim red light only (Northlight 36-6557, 15 lm, Clas Ohlson, Insjön, Sweden), which delivered less than 1 lux at floor level.

### Photoperiodic Treatment

All experiments were conducted from 30.09.2015 to 04.02.2016 and from 22.12.2017 to 08.04.2018. Birds hatched in the 2015/2016 season were exposed to three different photoperiodic treatments (Figure 1C). Initially, all birds were transferred into a photoperiod of L:D 12:12, which was gradually (1 h/day) decreased to L:D 6:18. The birds were subsequently kept in either L:D 6:18 (SP-group, *n* = 7) or were gradually (1 h/day) transferred into L:D 16:8 (LP), and then to LL (LP/LL-group, *n* = 8). Birds from the 2017 cohort were directly transferred from L:D 6:18 to DD (DD-group, *n* = 3). All birds were kept in their respective final light treatments until the end of the experiment. Photoperiodic treatments and exposure times for each bird can be found online at DataverseNO.^1^

### Core Body Temperature Recording

Core body temperature was measured using iButton temperature loggers (DS1922L, Maxim Integrated, San Jose, CA, United States; accuracy: ±0.5°C, resolution: ±0.0625°C). All iButtons were calibrated in a high precision water bath (model 6025, Hart Scientific, Pleasant Grove, UT, United States), the temperature of which was monitored by a factory-calibrated (Nordtec, Gothenburg, Sweden) Testo 925 thermometer with a type K thermocouple (Testo, PA, United States; birds from the 2015 cohort) or a high precision glass thermometer (birds from the 2017 cohort). Calibration was performed in 5°C increments between 35 and 45°C. This range covered the full range of core *T*_b_ shown by Svalbard ptarmigan over the course of the year (Nord and Folkow, 2018).

The calibrated iButtons were implanted into the abdominal cavity under gas anesthesia. Specifically, the birds were anesthetized with a 4% isoflurane air mix (Ref. No.: 9623, KDG Baxter, Deerfield, IL, United States) injected through an anesthetic facemask connected to an Ohmeda vaporizer (Ref. No.: 058294, BOC Health Care, Guildford, United Kingdom) and an isoflurane vaporizer (Vapor 2000, Ref. No.: ARXH-1225, Dräger, Lübeck, Germany). Surgery started as soon as the bird showed muscle relaxation and did not respond to a physical stimulus (pinching of the skin).

The place of incision, i.e., ventrocaudal from the sternum, was located, plucked of feathers and disinfected with 2% iodine (ref. no.: 332452, Sanivo Pharma AS, Oslo, Norway). The skin and muscle tissue were cut along the *linea alba* and the sterilized (70% EtOH) iButton was then inserted into the abdominal cavity. The muscle tissue was sutured with an absorbable 2-0 Polysorb string (Ref. No.: CL-811, Syneture, Dublin, Ireland) and was disinfected with 2% iodine. The skin was sutured with an absorbable 0 Dexon string (Ref. No.: 7232-61, Syneture, Dublin, Ireland) and again disinfected with 2% iodine. After surgery, the facemask was removed, and the bird was observed until it regained full consciousness. The birds were placed into their home cages as soon as they could stand unaided.

The iButtons recorded hourly *T*_b_ for the durations outlined online.^2^ Specifically, in the SP-group, the *T*_b_ of seven birds was recorded for 48 days (except for bird SP2 which was measured for 30 days). In the LP/LL-group, eight birds were recorded for 23 days under LP and 14 days under LL. In the DD-group, three birds were recorded for 83 days. All recordings were made at the full hour except for two birds in the SP-group, which recorded at half hour. At the end of the experiment, the implanted iButtons were recovered from euthanized birds and the data were downloaded using the Maxim Integrated software OneWireViewer (version 0.3.19.47).

### Activity Recording

Locomotor activity was recorded continuously as movements per minute using passive infrared sensors (HSP 1131, Panasonic, Kadoma, Japan). These were installed on homebuilt circuit boards and mounted on the cage doors. Data were collected for a subset of three birds per photoperiodic group (determined by the number of available recording devices), using an Actimetrics CL200 USB interface coupled to ClockLab data acquisition software Version 2.61 (Actimetrics, Wilmette, IL, United States).

We recorded activity for the experiment during 9 days in three birds in the LP/LL-group under LP, 14 days in three birds in the LP/LL-group under LL, and 66 days in three birds in the DD-group. In the SP-group we measured activity for 12, 18, and 31 days in three birds. Activity was recorded as counts per minute and normalized from 0 to 1 for each individual bird prior to analysis and plotting.

### Data Handling and Analysis

All graphs were plotted with GraphPad Prism 8 (Version 8.3.0, San Diego, CA, United States), except for the actograms, which were plotted using the ImageJ plugin ActogramJ (Schmid et al., 2011).

We plotted actograms for *T*_b_ and normalized activity for each bird over the whole experimental period. Actograms illustrate rhythmicity or the lack of it. All actograms were double-plotted to ease inspection. In a double-plotted actogram, one horizontal line represents 2 consecutive days (x-axis). Consecutive days are also plotted from top to bottom (y-axis). Normalized activity is displayed as bars of increasing heights between 0 and 1 on each line. This means that the higher the activity, the higher the bar. Low bars or the absence of bars indicate low activity and rest. Patterns or lack of rhythmicity can be observed by reading the actogram from top to button and by observing how phases of high and low activity relate to each other. We also adapted actograms to display *T*_b_ between 40 and 42°C to show *T*_b_ rhythmicity. Hence, *T*_b_ < 40°C is blank in the actogram, while temperatures > 40°C were plotted as bars of increasing height up to 42°C. Rhythmicity in these actograms was tested by calculating *χ*^2^-periodograms (Sokolove and Bushell, 1978) for 10 consecutive days for each bird in each light treatment. The 10-day period was chosen to coincide with reduced frequency of husbandry practices (see section “Bird Husbandry and the Effect on *T*_b_^”^). The *χ*^2^-periodogram algorithm calculated Q_p_ indices for each period between 1 and 30 h. Q_p_ follows a *χ*^2^ distribution, and values corresponding to a value *p* < 0.05 were considered statistically significant.

We also plotted *T*_b_ and activity as mean ± SD 24-h profiles for the SP and LP/LL group under LP. For each group, we calculated *T*_b_ peak and nadir for each respective light treatment. The peak and nadir means are based on maximum and minimum *T*_b_ of each day and each individual bird. We then compared the difference in daily *T*_b_ peak and nadir (henceforth “amplitude”) between photoperiod groups. For this, we used a mixed effects model fitted with restricted likelihood (lmer function in the lme4 package; Bates et al., 2014) using R version 4.0.0 (R Core Team, 2020) implemented in RStudio (version 1.3.959). Photoperiodic treatment was used as the explanatory variable, and a random intercept for bird ID was included to account for repeated measurements. Group estimates for the amplitude and comparisons between the groups were obtained using the emmeans R package (Lenth et al., 2018). Periods of transition between different photoperiods, and two birds from the SP-group which recorded at half hour, were excluded from this analysis.

Core body temperature and activity were also plotted together for a 5-day period and for three birds for each photoperiod. In the periods selected for this purpose, the birds were acclimatized to their respective photoperiod for at least a week and were undisturbed apart from normal husbandry. In order to analyze effects of photoperiod on dawn anticipation, we calculated mean *T*_b_ and activity from the 5-day periods for 5 h before light-on when *T*_b_ was at its minimum and for 1 h after light-on. The activity mean was calculated as the mean of the 10 min immediately before each *T*_b_ measurement. We defined dawn anticipation as nocturnal rise in *T*_b_ or activity that preceded the light-on signal. The 6-h period was analyzed by fitting a segmented linear regression (GraphPad 8) and we considered the break point of the segmented function (i.e., where the two regression segments meet) as the start of anticipatory rise in activity or *T*_b_. We plotted the data in Zeitgeber time (ZT), in which ZT 0 corresponds to the light-on signal.

### Bird Husbandry and the Effect on *T*_b_

Birds were monitored daily as part of routine husbandry. This might cause stress, which is known to cause increased *T*_b_ in birds (Cabanac and Guillemette, 2001; Nord and Folkow, 2019). This might affect rhythmicity analyses, especially in LL and DD. For this reason, we kept records of the timing of husbandry and tested how these visits affected *T*_b_. We defined three categories (husbandry, 1 h after husbandry, and no husbandry) and assigned the respective *T*_b_ reading to each category for birds under LL and DD. The *T*_b_ means for each bird and each category were compared using paired *t*-test (Graphpad 8). In addition, we plotted husbandry in LL and DD in form of actograms and conducted *χ*^2^-periodogram analyses on the rhythm of husbandry and on *T*_b_ recording of all birds under LL and DD.

## Results

**Figure 2:**
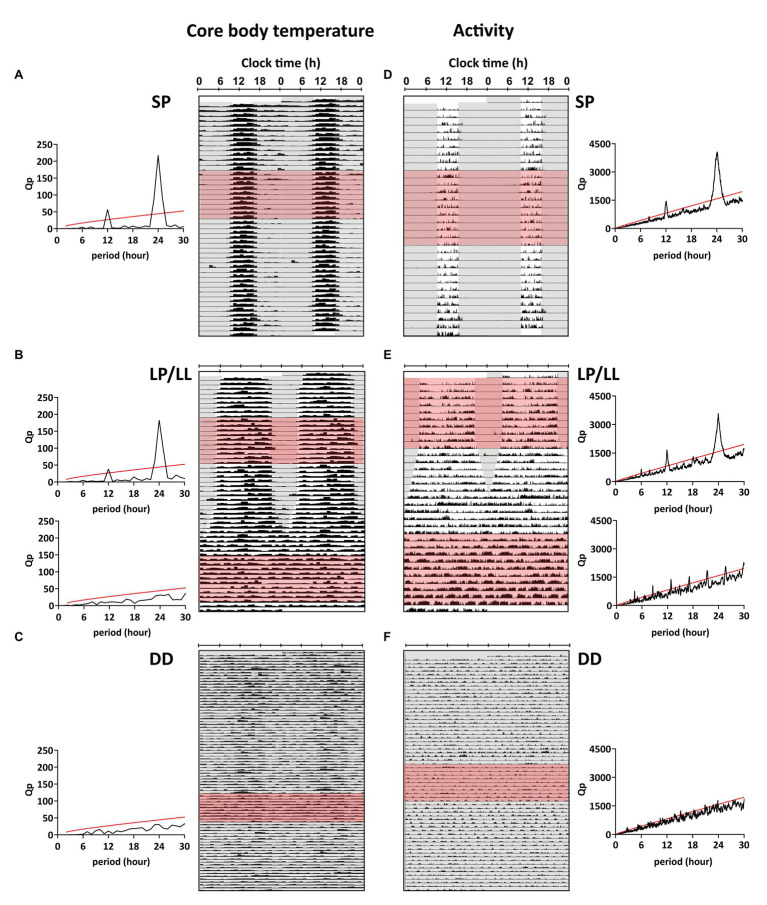
Representative double-plotted actograms for body temperature (*T*_b_) and activity. **(A-C)** *T*_b_ was plotted actogram-like between 40 and 42°C for representative birds from each group (bird IDs **A**: SP3, **B**: LP/LL11, **C**: DD3). **(D–F)** Actograms for normalized activity were plotted between 0 and 1 for representative birds from each group (bird IDs **D**: SP6, **E**: LP/LL15, **F**: DD2). *χ*^2^-periodograms were plotted for 10 consecutive days in each light treatment (red shading in actograms) and are displayed next to the respective recordings. Values above the red line indicate that the cycle period was significant (*p* < 0.05). Additional actograms and periodograms can be found in Supplementary Figures S1–S3.

Svalbard ptarmigan held under SP and LP displayed clear daily rhythms in *T*_b_ with a 24-h period (*p* < 0.05 by *χ*^2^-periodogram), while birds under LL and DD showed no significant rhythmicity in *T*_b_ for a chosen 10-day period (*p* > 0.05 for all periods by *χ*^2^-periodogram; Figures 2A–C). Husbandry caused stress related increase in *T*_b_ (Supplementary Figures S5, S6) and the 10-day periods (especially under DD) were chosen to coincide with less and randomized husbandry (Supplementary Figure S6).

**Figure 3:**
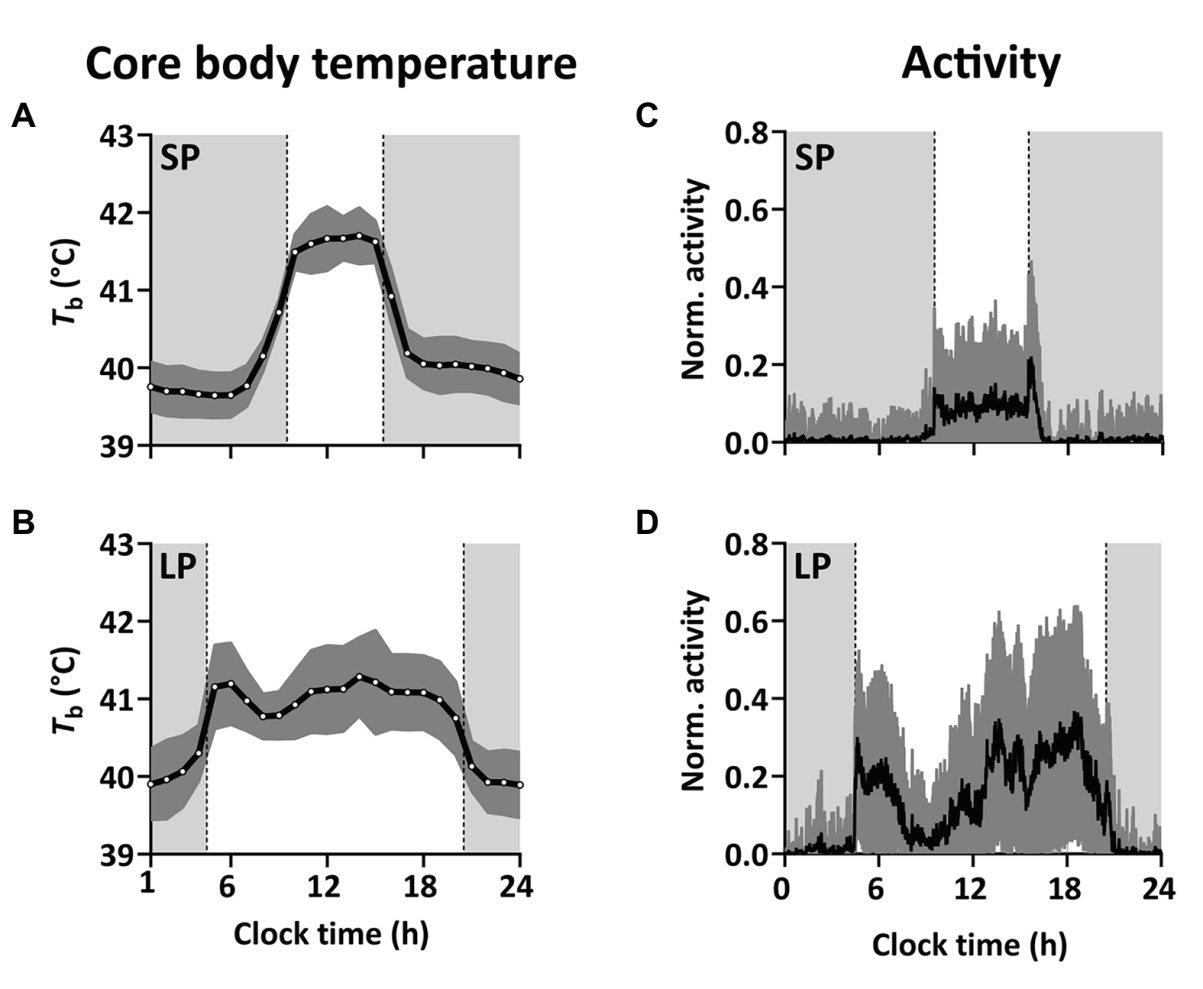
Diel variation in *T*_b_ and activity in short and long photoperiod. **(A,B)** Mean ± SD *T*_b_ over the course of 24 h (01:00 to midnight) in SP (based on 222 × 24-h recordings from five birds) and LP (based on 184 × 24-h recordings from eight birds). *T*_b_ was measured every hour throughout the experiment. **(C,D)** Mean normalized activity ± SD over the course of 24 h (midnight to midnight) in SP (61 × 24-h recordings from three birds) and LP (27 × 24-h recordings from three birds). Light gray shadowing in the panels indicate periods of darkness and dark gray indicates SD.

**Figure 4:**
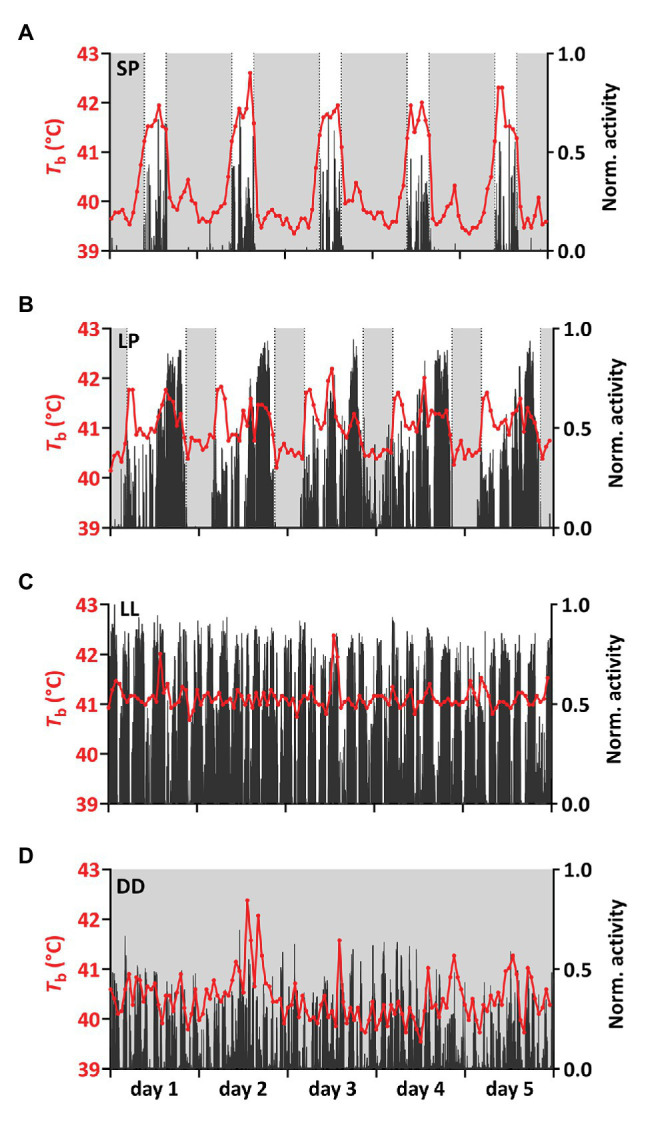
Representative time series for *T*_b_ and activity. **(A–D)** *T*_b_ (red) was plotted together with normalized activity (black) for 5 consecutive days for one representative bird per experimental treatment (bird IDs **A**: SP6, **B,C**: LP/LL13, **D**: DD2). Light gray shadings indicate periods of darkness. Additional time series can be found in Supplementary Figure S4.

The *T*_b_-rhythm under SP and LP is defined by decreased *T*_b_ during the dark-phase and increased *T*_b_ during the light-phase, which is expressed either as a single peak in SP (Figures 3A, 4A) or as a morning and one or several “afternoon peaks” in LP (Figures 3B, 4B). Birds under SP and LP displayed *T*_b_ amplitudes of 2.52 ± 0.15°C and 2.27 ± 0.12°C, respectively (estimate ± SE by mixed model analysis), while birds under LL and DD showed smaller differences between peak and nadir (*p* < 0.001 by mixed model analysis) with 1.46 ± 0.12°C and 1.30 ± 0.19°C, respectively. The amplitudes between SP vs. LP (*p* = 0.532) and LL vs. DD (*p* = 0.891) did not differ significantly. Results from the mixed model analyzing the amplitude are presented in Table 1.

**Table 1:** Differences in body temperature amplitude (i.e., the difference between the daily core *T*_b_ peak and *T*_b_ nadir; °C) under different photoperiods.

	Estimate (SE)	LR	*p*
**Model**			
Intercept	1.30 (0.17)		
Treatment		61.584	<0.001
Constant darkness (DD)	1.30 (0.19)		
Constant light (LL)	1.46 (0.12)		
Long photoperiod (LP, L:D 16:8)	2.27 (0.12)		
Short photoperiod (SP, L:D 6:18)	2.52 (0.15)		
**Contrast**			
DD vs. LL	−0.16 (0.22)		0.891
DD vs. LP	−0.96 (0.22)		<0.001
DD vs. SP	−1.22 (0.24)		<0.001
LL vs. LP	−0.81 (0.17)		<0.001
LL vs. SP	−1.06 (0.19)		<0.001
LP vs. SP	−0.26 (0.19)		0.532

Estimates (±SE), likelihood ratios (LR) and *p*-values for the differences between photoperiodic treatments. Data were tested using a linear mixed effects model with bird ID as a random intercept. *p*-values and group estimates were obtained and compared using the emmeans package in R.

Svalbard ptarmigan under SP and LP displayed clear 24-h rhythmicity in activity (*p* < 0.05 by *χ*^2^-periodogram; Figures 2D,E) with high activity in the light-phase and low activity in the dark-phase (Figures 3C,D). Similar to *T*_b_, birds under LP also displayed two distinct activity peaks during the light phase (Figure 3D). Birds under LL and DD showed various significant periods in activity between 1 and 30 h (*p* < 0.05 by *χ*^2^-periodogram; Figures 2E,F).

**Figure 5:**
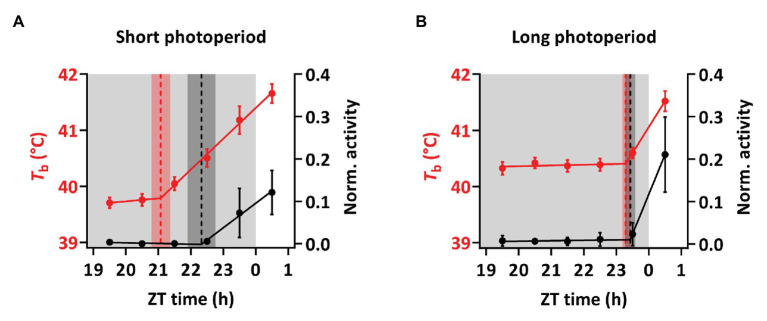
Anticipatory rise in *T*_b_ based on segmental regression breakpoints. Hourly means of *T*_b_ (red) and activity (black) 5 h before and 1 h after the light-on signal, given in Zeitgeber time (ZT). **(A)** *T*_b_ in birds under SP was rising 2 h 56 min before the light-on signal while activity rises 1 h 40 min before light-on. **(B)** In birds under LP *T*_b_ increased 43 min before light-on, while activity rose 34 min before the light-on signal. The data correspond to the measurement in Figure 4 and Supplementary Figure S4 and are displayed as mean ± 95% CI. Dotted lines indicate segmented regression breaking points and the shading shows the corresponding SD. Light gray shadings indicate periods of darkness.

Birds under SP showed a significant nocturnal increase in *T*_b_ preceding the light-on signal and the rise in activity (Figures 4, 5). Under SP, both activity and *T*_b_ increased in the 5-h period immediately preceding the light-on signal. However, *T*_b_ rose 3 h before light-on [segmented regression breakpoint: ZT 21:04 ± 00:17 (hh:mm ± SD)] whereas activity increased 1 h 40 m before (breakpoint: ZT 22:20 ± 00:26). In LP, birds showed increased *T*_b_ and activity starting around half an hour before light-on (breakpoint for *T*_b_: ZT 23:17 ± 00:06; and for activity: ZT 23:26 ± 00:09).

We also observed that SP-birds often expressed a small increase in *T*_b_ in the dark-phase around 11 h after the last light-on switch (ZT 11:05 ± 1:00, mean ± SD based on analysis in Figure 4 and Supplementary Figure S4). The nocturnal peak was similar to the expression of the “afternoon peak” in LP-birds that occurred 9 h after the light-on signal (ZT 8:51 ± 1:11).

## Discussion

In this study, we measured *T*_b_ and activity in Svalbard ptarmigan under photoperiods that were representative of those experienced during their annual cycle in the wild. All recorded *T*_b_ fell within the reported range of birds (Prinzinger et al., 1991) and were comparable to previous measurements of *T*_b_ during subjective daytime in Svalbard ptarmigan (Nord and Folkow, 2018). In SP and LP, birds showed pronounced rhythms in *T*_b_ with high temperatures during the light-phase and low temperature during the dark-phase. In LL and DD, the difference between maximal and minimal *T*_b_ within a 24-h period was attenuated and our periodogram analysis suggests weakened circadian control over *T*_b_ rhythms under these constant photic conditions. Occasionally, birds in LL and DD continued to show transient peaks in *T*_b_, which are reminiscent of sustained rhythmicity. However, these peaks coincided with visits for husbandry and we ascribe the observation to consequences of a stress-related increase in *T*_b_ (Cabanac and Guillemette, 2001; Nord and Folkow, 2019).

Activity was also rhythmic with a 24-h period in SP and LP, which was not sustained in LL and DD. This is in accordance with previous studies on activity rhythms in Svalbard ptarmigan (Stokkan et al., 1986; Reierth and Stokkan, 1998). Instead of a clear 24-h rhythm, birds under LL and DD showed various significant periods between 1 and 30 h. Especially birds under LL appeared to express ultradian activity rhythms with a period of ca. 4 h (Figure 2E) of which subsequent peaks in the periodogram could be subharmonics to the fundamental ultradian period. In the absence of an environmental light-dark cycle, the ultradian rhythm might reflect foraging activity and subsequent rest in Svalbard ptarmigan. At this stage, we can only speculate if this activity pattern is under endogenous control of an ultradian oscillator (Bourguignon and Storch, 2017) or if it is produced by the interplay of hunger and satiety.

Core body temperature rose in anticipation to the light-on signal and, in birds under SP, prior to rises in activity. This suggests, firstly, that, as in most other endothermic animals, *T*_b_ is a distinct endogenous feature and not only a consequence of activity (Menaker, 1959; Aschoff, 1983; Refinetti and Menaker, 1992). Secondly, it suggests that the *T*_b_-cycle in Svalbard ptarmigan is controlled by a time-measuring system, which accurately anticipates the light-on signal. In nature, this anticipatory rise in *T*_b_ might ensure optimal bodily function at the start of the active phase. In mammals, cycles in *T*_b_ might be utilized by the central circadian system to impose its rhythm on peripheral tissues (Brown et al., 2002; Buhr et al., 2010). It is possible that Svalbard ptarmigan (and other birds) use *T*_b_ in the same manner to ensure synchronized physiology in the short periods of light-dark cycles in-between the long stretches of polar day and polar night. Due to the absence of measurable circadian rhythms in *T*_b_ and activity in LL and DD, we propose that the central circadian system of Svalbard ptarmigan either uncouples from its output, or dampens in rhythmicity, when there is no periodic environmental synchronization (Bloch et al., 2013). This might ensure around-the-clock foraging without endogenous restraints during the polar day and polar night. However, we cannot exclude the possibility that under natural conditions Svalbard ptarmigan still express rhythms in *T*_b_ during the polar day and polar night due to entrainment to other photic or non-photic cues (Long et al., 2005; Ashley et al., 2012).

We also observed transient increases in *T*_b_ in the dark-phase of birds under SP. This could reflect nocturnal digestive activity (Rashotte et al., 1997). Alternatively, this observation might be further support for a circadian drive in *T*_b_ under light-dark cycles, in which case the transient nocturnal *T*_b_-peak in SP would correspond to the “afternoon peak” seen in LP birds.

Our findings suggest that Svalbard ptarmigan are using a circadian system under SP and LP to control their *T*_b_. Under prolonged LL and DD, this circadian control of *T*_b_ and activity seems to weaken. They can, therefore, utilize the benefits of a circadian system during times of a rhythmic environment but are able to escape its restrictions in constant photic conditions. Instead of a circadian rhythm, Svalbard ptarmigan show signs of ultradian rhythmicity in activity, especially under LL, but we cannot resolve if this rhythm is endogenous or produced by the interaction of hunger and satiety. Future research should aim to elucidate how Svalbard ptarmigan achieve their duality of circadian organization and how ultradian rhythmicity is controlled.

## Data Availability Statement

The datasets presented in this study can be found in online repositories. The names of the repository/repositories and accession number(s) can be found in the article/Supplementary Material.

## Ethics Statement

The animal study was reviewed and approved by the Norwegian Food Safety Authority (Mattilsynet).

## Author Contributions

DA, DH, and GW: conceptualization and project administration. DA: data curation and writing – original draft. DA, AN, and GW: formal analysis, investigation, and visualization. DH and GW: funding acquisition and supervision. DA, AN, DH, and GW: methodology and writing – review and editing. AN, DH, and GW: resources. AN: software. AN and GW: validation. All authors contributed to the article and approved the submitted version.

### Conflict of Interest

The authors declare that the research was conducted in the absence of any commercial or financial relationships that could be construed as a potential conflict of interest.

## Footnotes

^1^https://doi.org/10.18710/XLDXQ3

^2^https://doi.org/10.18710/XLDXQ3
