# Supplementary material for: Body Temperature and Activity Rhythms Under Different Photoperiods in High Arctic Svalbard ptarmigan (Lagopus muta hyperborea)
Source: Front Physiol. 2021 Mar 8;12:633866. doi: 10.3389/fphys.2021.633866 (PMC7982588; doi:10.3389/fphys.2021.633866)
Supplement: Supplementary file 1 [file Data_Sheet_1.pdf]

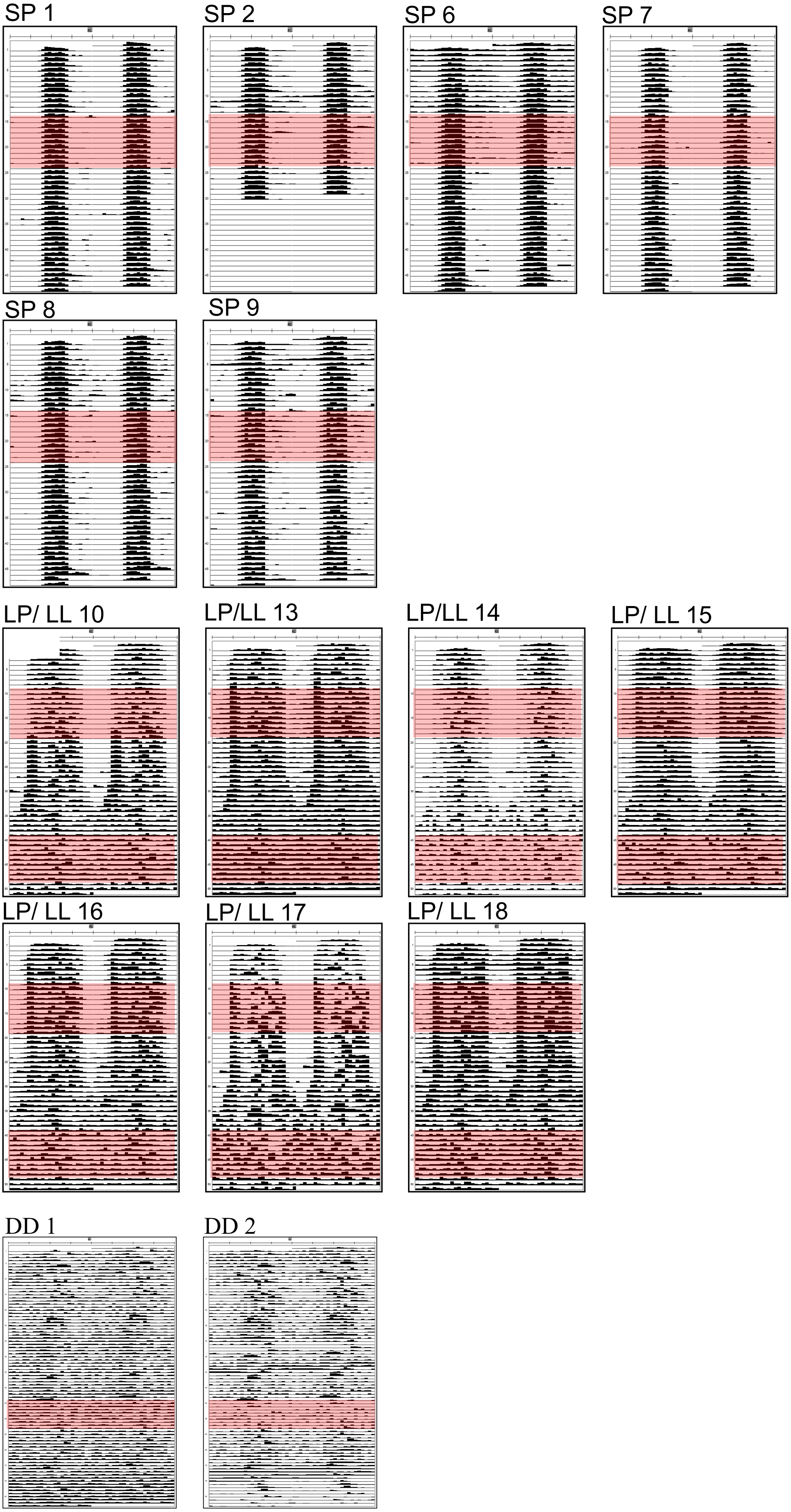

Figure S1 | Additional T_b-actograms. T_b was plotted actogram-like between 40 and 42°C for all birds from each group (representitive birds from the main text exceptd). T_b-actograms are labelled according to the group and bird-ID and red shadings indicate analyses by $\chi 2$-periodograms (Figure S2).

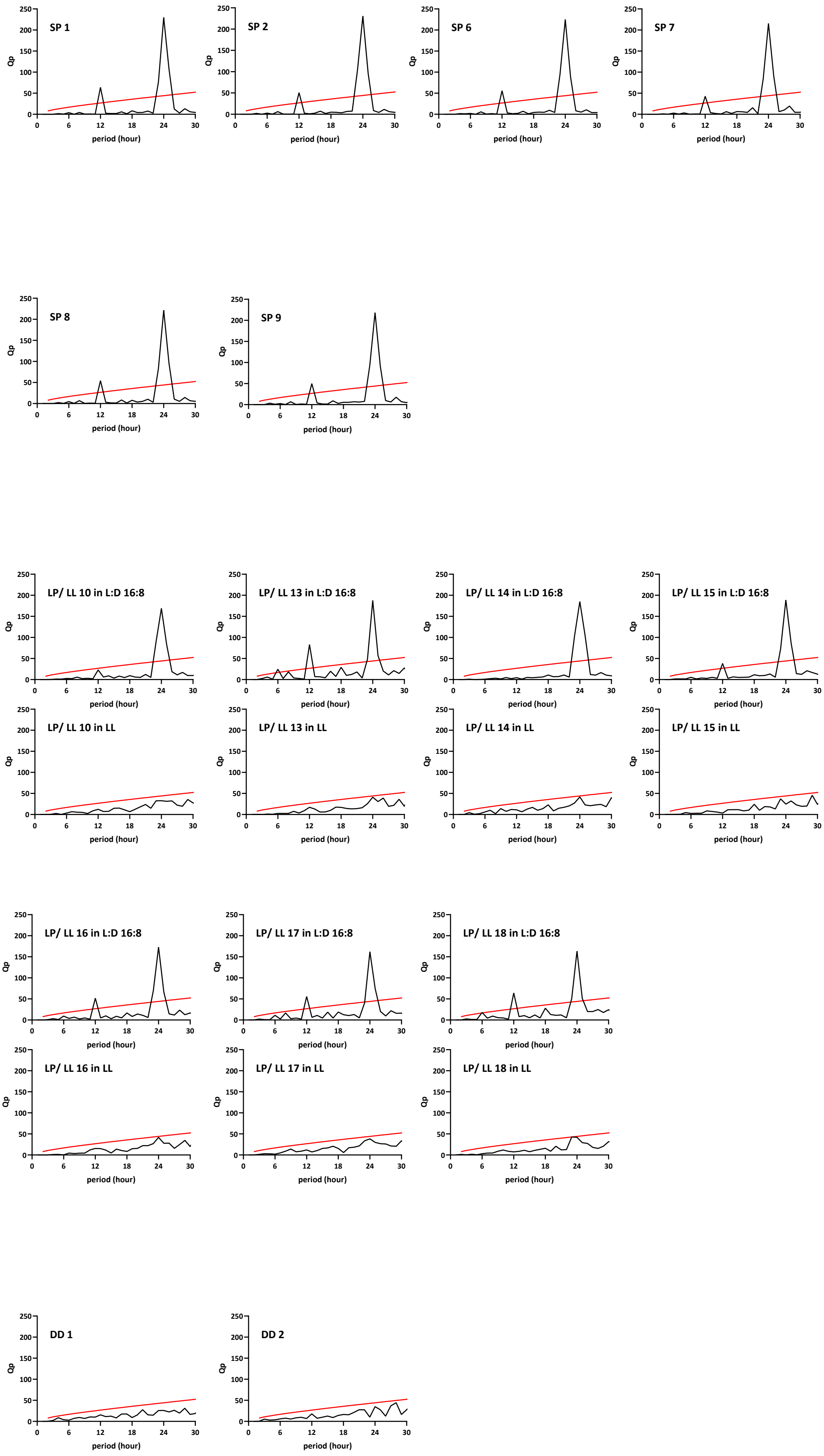

Figure S2 | Additional χ^2-periodograms for T_b-actograms of Figure S1. χ^2-periodograms were plotted for ten consecutive days in each light treatment. Values above the red line indicate significant periods of the cycles ($p < 0.05$). Bird-IDs correspond to Figure S1.

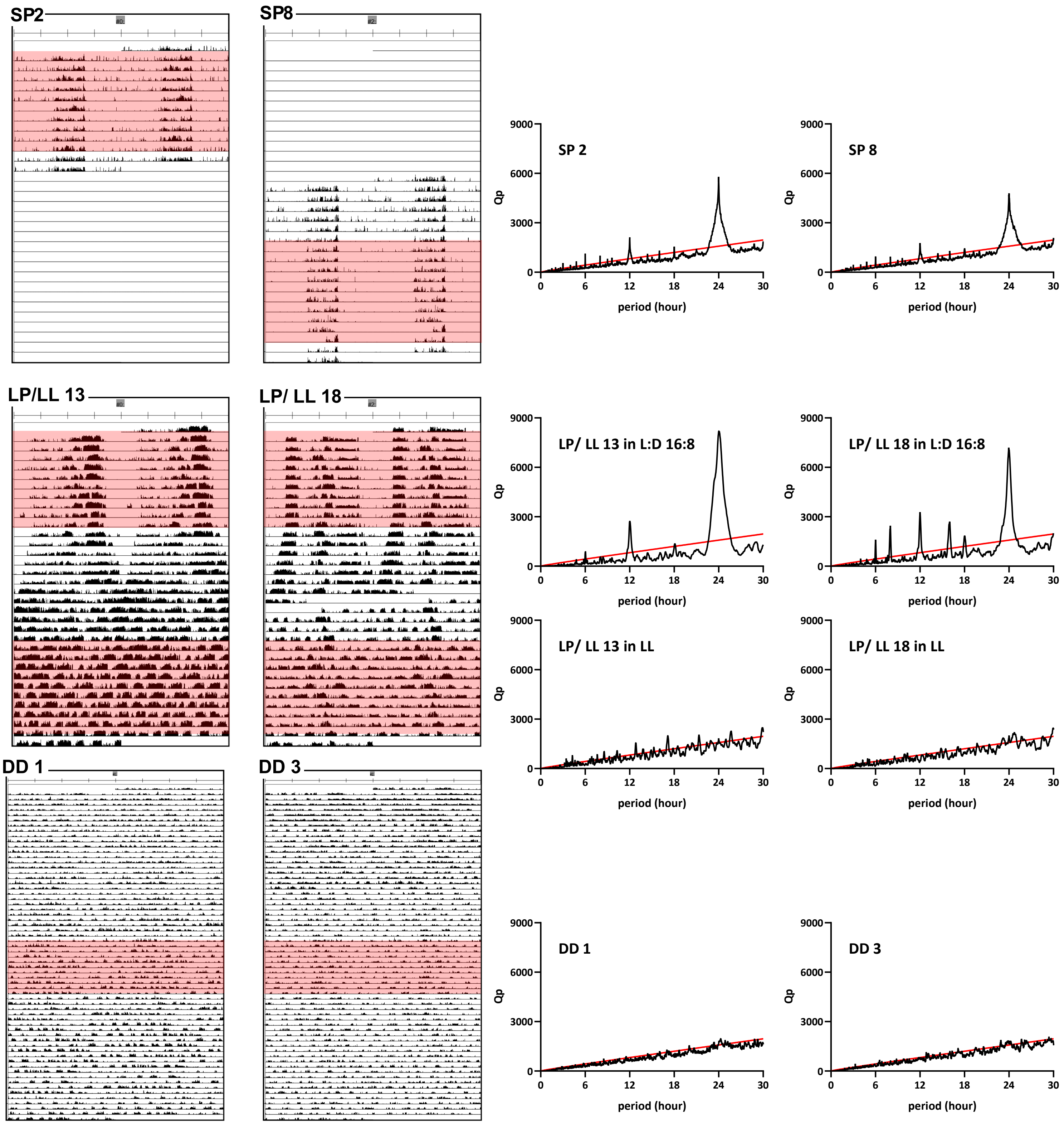

Figure S3 | Additional actograms for normalized activity. All actograms were double plotted for all birds from each group (representitive birds from the main text exceptd). Actograms are labelled according to the group and bird-ID. Red shadings indicate analyses by χ2-periodograms. Corresponding χ2-periodograms were plotted for ten consecutive days in each light treatment (red shading). Values above the red line indicate significant periods of the cycles ($p < 0.05$).

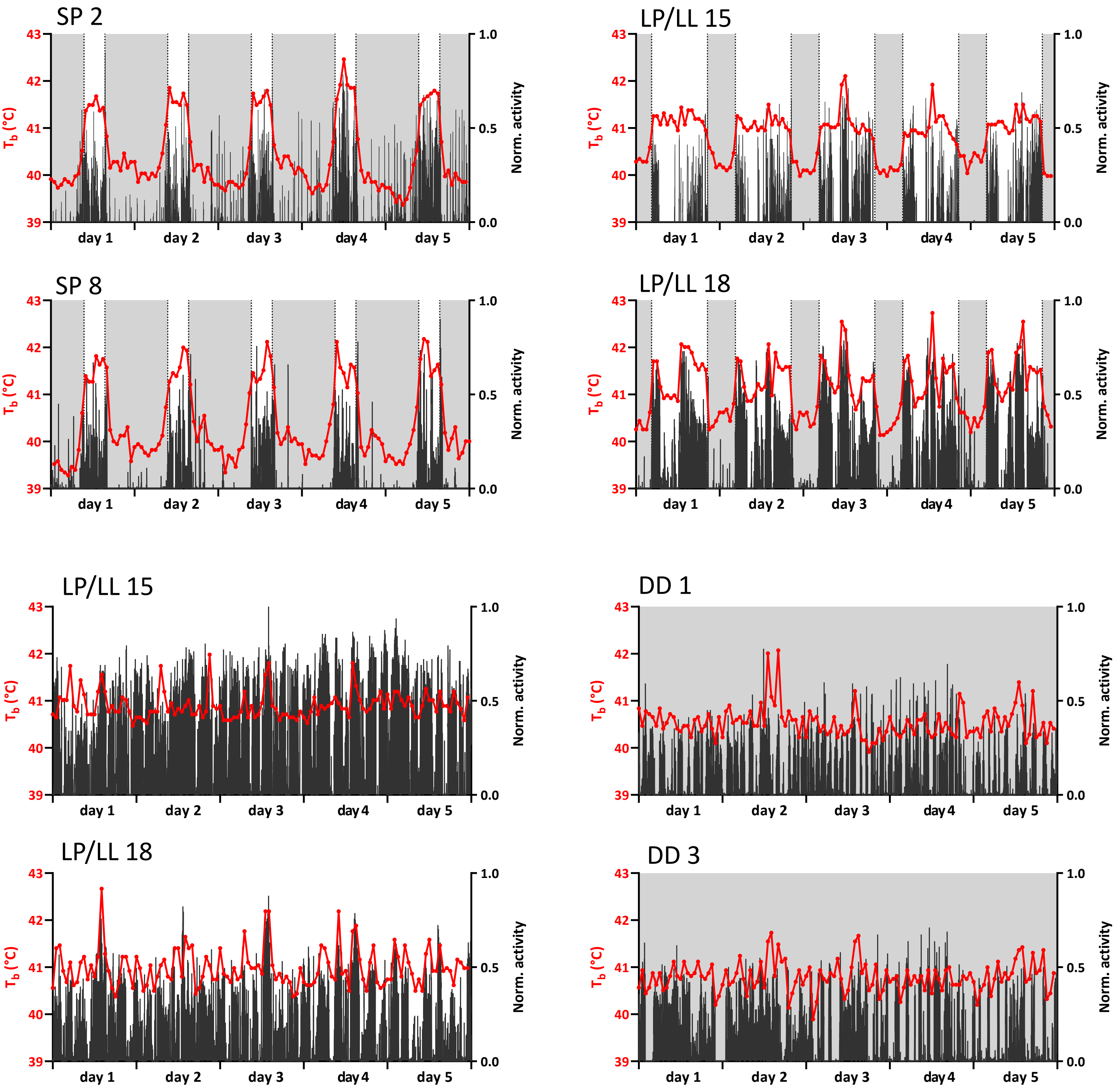

Figure S4 | Additional time series for T_b and activity. T_b (red) was plotted with normalized activity (black) for five consecutive days for two birds from each experimental treatment (bird-IDs are shown in the graph). Light grey shadings indicate periods of darkness.

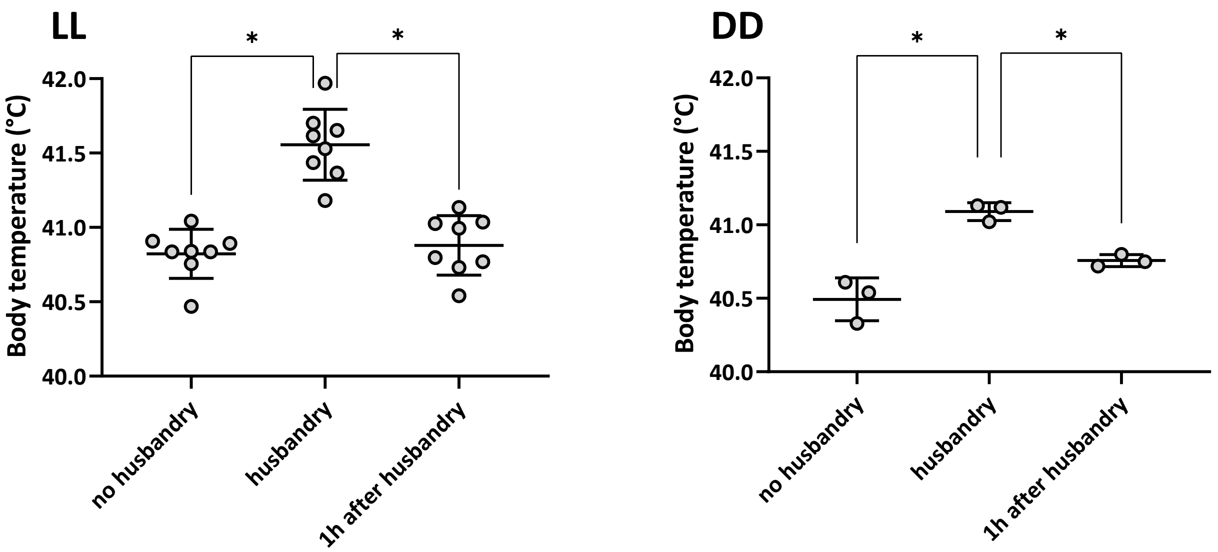

Figure S5 | The effect of husbandry on body temperature (T_b) in constant light (LL) and constant darkness (DD). The mean T_b of each bird under each condition (no husbandry, husbandry and 1 h after husbandry) was compared using paired *t*-tests. T_b was different between 'no husbandry' and 'husbandry' (LL: $p < 0.0001$, $t = 11.86$, df = 7 | DD: $p = 0.0256$, $t = 6.13$, df = 2) and between 'husbandry' and '1 h after husbandry' (LL: $p < 0.0001$, $t = 9.76$, df =7 | DD: $p = 0.0288$, $t = 5.77$, df = 2). There was no significant difference between 'no husbandry' and '1 h after husbandry' (LL: $p = 0.2353$, $t = 1.30$, df = 7 | DD: $p = 0.0987$, $t = 2.94$, df = 2). Data is displayed as mean ± SD.

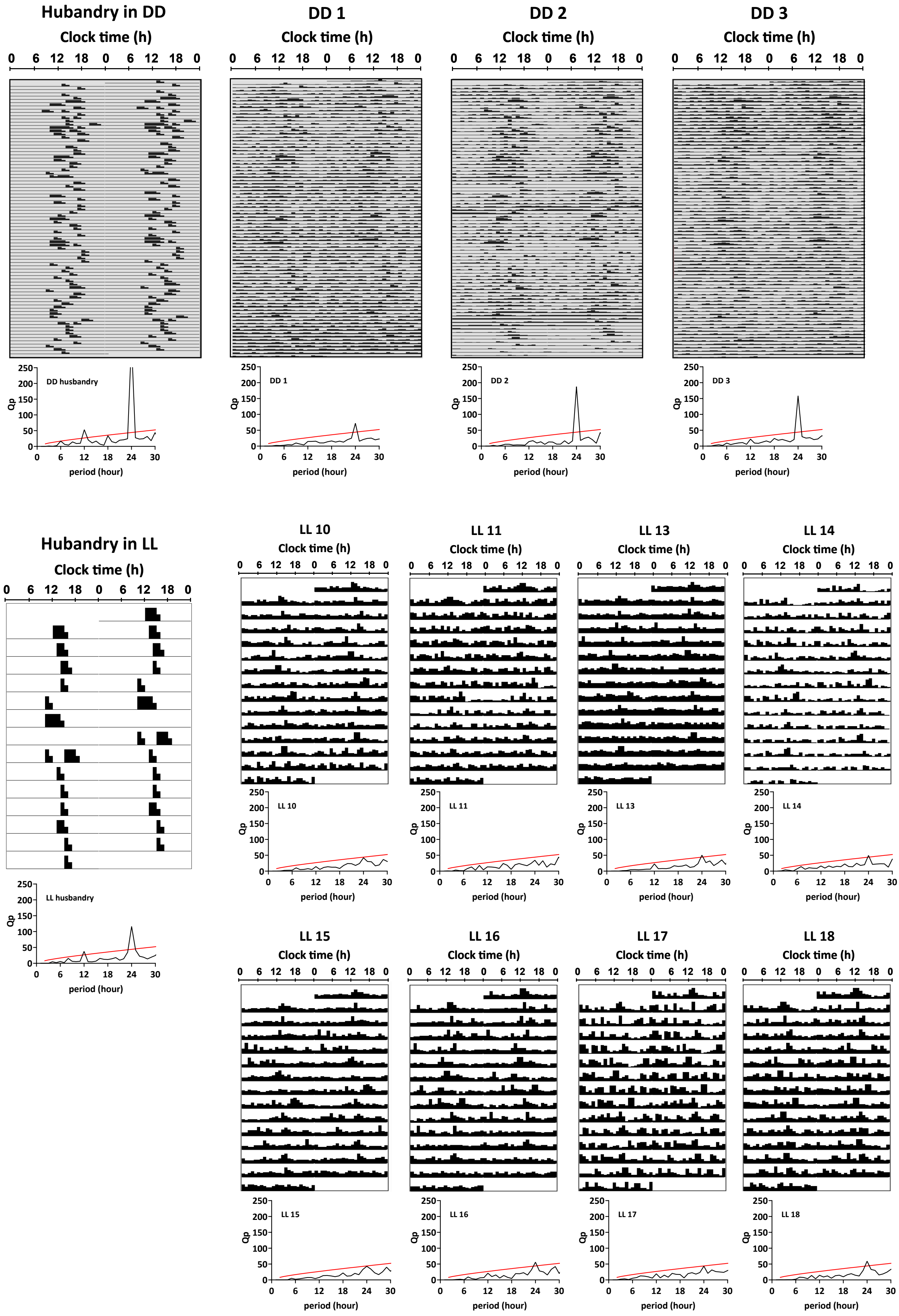

Figure S6 | Actograms of T_b and husbandry in LL and DD and their respective χ^2-periodograms . Husbandry is shown in actogram form with black bars indicating the hour of husbandry (high bar) and 1 h thereafter (low bar). T_b actograms of all birds under LL and DD are displayed with their respective ID next to the husbandry actograms. χ^2-periodograms were calculated for the whole range and values above the red line indicate significant periods of the cycles ($p < 0.05$).
